# The Tumor-Specific Immune Landscape in HPV+ Head and Neck Cancer

**DOI:** 10.3390/v15061296

**Published:** 2023-05-31

**Authors:** Jacob P. Conarty, Andreas Wieland

**Affiliations:** 1 Department of Otolaryngology, The Ohio State University, Columbus, OH 43210, USA; 2 Pelotonia Institute for Immuno-Oncology, The Ohio State University Comprehensive Cancer Center-Arthur G. James Cancer Hospital and Richard J. Solove Research Institute, The Ohio State University, Columbus, OH 43210, USA; 3 Biomedical Sciences Graduate Program, The Ohio State University, Columbus, OH 43210, USA; 4 Department of Microbial Infection and Immunity, The Ohio State University, Columbus, OH 43210, USA

**Keywords:** human papillomavirus, HPV, head and neck cancer, immuno-oncology, tumor-specific B cells, tumor-specific T cells, HPV-specific antibodies

## Abstract

Human papillomaviruses (HPVs) are the causative agent of several anogenital cancers as well as head and neck cancers, with HPV+ head and neck squamous cell carcinoma (HNSCC) becoming a rapidly growing public health issue in the Western world. Due its viral etiology and potentially its subanatomical location, HPV+ HNSCC exhibits an immune microenvironment which is more inflamed and thus distinct from HPV-negative HNSCC. Notably, the antigenic landscape in most HPV+ HNSCC tumors extends beyond the classical HPV oncoproteins E6/7 and is extensively targeted by both the humoral and cellular arms of the adaptive immune system. Here, we provide a comprehensive overview of HPV-specific immune responses in patients with HPV+ HNSCC. We highlight the localization, antigen specificity, and differentiation states of humoral and cellular immune responses, and discuss their similarities and differences. Finally, we review currently pursued immunotherapeutic treatment modalities that attempt to harness HPV-specific immune responses for improving clinical outcomes in patients with HPV+ HNSCC.

## 1. Introduction

The incidence of human papillomavirus (HPV)-associated head and neck squamous cell carcinoma (HNSCC) has dramatically increased over the last few decades, with currently approximately 18,000 new cases being diagnosed annually in the United States (US) [1,2,3,4]. Notably, the number of new HPV+ HNSCC cases is estimated to exceed 30,000 per year by 2030 [5]. At present, HPV+ HNSCC accounts for approximately 90% of all oropharyngeal cancers and 40% of all HPV-associated cancers in the US [4]. As a comparison, approximately 11,000 new cases of cervical cancer are diagnosed annually in the United States, accounting for about 30% of all HPV-associated cancers [6]. While the rates of cervical cancer in the US have continuously decreased over the last two decades, HPV+ oropharyngeal cancer cases in men continue to steadily climb at 2.7% per year [7].

While both HNSCC and cervical cancer develop from high-risk HPV subtypes, there is a noticeable difference in the presence of individual HPV types in these two malignancies. HPV16 is the most prevalent high-risk HPV type and accounts for the plurality (30–50%) of HPV+ cervical cancers [8,9] but the vast majority (>80%) of HPV-associated cancers in the oropharynx [10,11,12,13,14,15,16]. Notably, while HPV18 is the second most common HPV type in cervical cancer, accounting for about 16% [17], HPV18 and other high-risk types such as HPV31, HPV33, HPV35 and HPV52 only account for a minor fraction of HPV+ HNSCC. Importantly, the factors contributing to the striking dominance of HPV16 among other high-risk HPV types in HNSCC are ill understood, thus warranting additional investigation.

The continuous rise in the incidence of HPV+ HNSCC is not solely restricted to the US but has also been observed in other parts of the Western world. For example, the incidence of HPV+ oropharyngeal cancer in the United Kingdom doubled between 2002 and 2011 [18,19]. Importantly, while the Western world experiences a dramatic rise in HPV+ oropharyngeal cancer, which is now dominating in terms of cases over “traditional” tobacco- and alcohol-associated cancers, this trend is also seen, albeit to a lesser extent, globally. While rates of HPV+ HNSCC in China are similar to the United States [20], certain regions of Africa exhibit much lower rates [21]. Although HPV+ oropharyngeal cancer has now replaced cervical cancer as the leading HPV-associated cancer in the Western world, which is mostly due to regular cervical cancer screenings and prophylactic HPV vaccination efforts, globally, cervical cancer still vastly outnumbers HPV+ oropharyngeal cancer with 530,000 versus 29,000 newly diagnosed cases per year, respectively [22].

The major capsid protein L1, which self-assembles into virus-like particles (VLPs), forms the basis of all prophylactic HPV vaccines [23]. Recombinant VLPs consisting of L1 closely resemble the native HPV virions and are highly immunogenic, thus explaining the success of this vaccination approach [23,24,25]. In the US, three HPV vaccines have been licensed to date, with the nonavalent vaccine GARDASIL^®^9 being the solely distributed HPV vaccine in the US since 2016. The nonavalent vaccine contains VLPs of nine different HPV types, including low-risk types mostly associated with genital warts (HPV6 and 11) and the most common high-risk types (HPV16, 18, 31, 33, 45, 52, and 58). Prophylactic HPV vaccines have been reported to induce long-lasting immune responses, with responses being detectable for at least a decade [26,27,28]. While there is no established correlate of protection, which has been partly attributed to the high immunogenicity and efficacy of these vaccines, neutralizing antibodies are thought to be the main mechanism of protection [29].

Prophylactic HPV vaccines induce vigorous antibody responses, which have been characterized regarding magnitude, isotype composition, and neutralizing capacity in circulation as well as in cervical/vaginal secretions [30,31,32,33,34]. Both HPV-specific IgG and IgA antibodies are detectable in plasma after vaccination, with IgG antibodies dominating the response. Importantly, HPV-specific IgA and IgG antibodies are also detectable in cervical and vaginal secretions, with IgG being the most prominent isotype, akin to plasma [30]. In contrast to intramuscular vaccination, natural HPV infection results in lower IgG but comparable IgA plasma antibody titers [35,36], highlighting the important role of exposure route and potentially inflammatory context in antibody isotype switching. Notably, mucosal secretions of infected individuals exhibit higher IgA titers compared to vaccinated individuals [35], likely due to the retention of IgA+ plasma cells in mucosal sites of antigen encounter and thus in situ IgA secretion [37]. Furthermore, with respect to HPV+ HNSCC, HPV-specific IgG antibodies have also been detected in the saliva of vaccinees [38], supporting the notion that these vaccines are likely protective against oral infection as well. Indeed, multiple studies assessing oral HPV infections reported markedly reduced infection rates in vaccinees [39,40,41], highlighting that the currently available vaccines have the potential to reduce the incidence of HPV+ HNSCC in the future by preventing the initial infections.

Prophylactic HPV vaccination, first introduced in the US in 2006 and initially recommended only for females, is now being highly advocated for both women and men. While cervical cancer rates in the US continuously decreased over the past decades through the widespread implementation of cervical cancer screening [42], data highlighting the effectiveness of the HPV vaccine in preventing invasive cervical cancer are now slowly emerging as long lead times between the initial infection and cervical cancer diagnosis hamper the ready assessment of vaccine effectiveness [43]. For HPV+ HNSCC, however, which is a predominantly male disease affecting three- to six-fold more men than women [44,45], beneficial effects from HPV vaccination efforts have not yet come to fruition. The divergent incidence trends for HPV+ HNSCC and cervical cancer in the US are likely multifactorial. First, in contrast to cervical cancer, routine screening approaches for HPV+ HNSCC are lacking. Second, the approval of the HPV vaccine for men was delayed and initially restricted to younger individuals (age 9–21), except for risk groups (up to age 26) [46,47]. Third, the initial vaccine uptake in men was tepid. However, the current male vaccination rate of 60–70% is about to finally close the gender gap [7,48]. Finally, the long lag phase between initial infection and development of overt HPV+ HNSCC, which is estimated to be about 10–30 years based on individuals typically contracting HPV in their early 20s and the average age at HPV+ HNSCC diagnosis (mid 50s) and further supported by seroconversion as well as “mutational clock” studies [49,50,51], substantially delays the observance of vaccine benefits. Due to the above-mentioned factors, the current HPV+ HNSCC epidemic is predicted to last past 2060, with current vaccination efforts only expected to slowly curb the incidence of HPV+ HNSCC from 2045 onwards [52].

## 2. HPV+ HNSCC: A Distinct Form of HNSCC and HPV-Associated Cancer

HPV+ HNSCC is distinct from HPV-negative HNSCC in several important aspects such as therapy response rates, patient characteristics, mutational load, and subanatomical location. Various factors most likely contribute to the higher response rates of HPV+ HNSCC, compared to HPV-negative HNSCC, to standard-of-care regimens such as chemotherapy, radiation, and surgery, resulting in 5-year recurrence-free survival rates of 80–85% [53]. The prognostic significance of HPV status in HNSCC is also reflected in the recently updated 8th edition of the staging guidelines of the American Joint Committee on Cancer (AJCC), which effectively resulted in a major “downstaging” of HPV+ HNSCC as it incorporates the common presentation of lymph node metastases and restricts stage IV diagnoses to patients with distant metastases [54]. An important defining characteristic of patients with HPV+ HNSCC recognized during the early days of the HPV+ HNSCC epidemic is that this disease mostly affects a younger and healthier population of males, who are less likely to have a smoking history [53]. The overall younger age and better health status of patients with HPV+ HNSCC compared to patients suffering from traditional alcohol- and smoking-associated cancers most likely contributes to the improved response rates. However, recent data suggest that the age of patients with HPV+ HNSCC at time of diagnosis is increasing, thus closing the age gap to HPV-negative HNSCC [55,56].

The excellent response rates of HPV+ HNSCC patients and their overall better health status have triggered substantial efforts, referred to as treatment de-intensification or de-escalation, which are aimed at reducing treatment intensity with the overall goal of minimizing treatment-associated morbidities while maintaining high cure rates. Traditional standard-of-care therapies often induce significant morbidities such as nerve damage, salivary gland damage, tooth damage, and dysphagia, thus substantially reducing the quality of life of these relatively young and healthy individuals [57,58]. Currently explored de-escalation strategies involve omittance of chemotherapy, reduced radiation doses, and transoral robotic surgery (TORS) for minimally invasive, tissue-sparing resections, as well as incorporation of immunotherapeutic treatment modalities [59,60]. Notably, the first randomized de-escalation trials showed a clear detriment in survival in a subset of patients when chemotherapy was omitted or simply substituted with cetuximab [61,62]. Overall, these studies highlight the lack of reliable biomarkers to faithfully distinguish patients who would benefit from treatment de-escalation from patients who are at higher risk of recurrence and would potentially even benefit from increased treatment intensity.

Due to its viral etiology, the genetic landscape of HPV+ HNSCC substantially differs from HPV-negative HNSCC, which is mostly caused by prolonged exposure to chemical carcinogens as a result of smoking, alcohol use, and chewing of tobacco or betel nut products [63,64,65]. The divergent genetic landscapes in HPV+ and HPV-negative HNSCC are the result of two distinct oncogenic pathways, with HPV-negative cancers frequently displaying mutations of several tumor suppressors such as p53, which is mutated in the vast majority of HPV-negative HNSCC [66,67,68]. In contrast, HPV+ HNSCC rarely exhibits p53 or other common driver mutations. HPV+ HNSCC is, instead, driven by the expression of the viral oncoproteins E6 and E7 that inactivate the cellular tumor suppressors p53 and pRb, respectively, and are thus required for the persistence of HPV-associated cancers [69,70]. Consistent with the reduced dependence on cancer-driving mutations, two studies using whole-exome sequencing demonstrated that HPV+ HNSCC exhibits a significantly lower mutational burden than HPV-negative HNSCC [66,67]. However, a study examining 617 cancer-associated genes in a larger cohort of 120 patients with HNSCC, including 51 HPV+ cases, did not find significant differences in mutational loads [68]. These discrepant findings might be the result of different sequencing methodologies (whole-exome vs. selected gene set) as well as the composition of the HPV+ HNSCC populations at hand, as the latter study reported a strikingly higher mutational burden in patients with HPV+ HNSCC who had a history of heavy tobacco use. Nevertheless, independent of the mutational burden, HPV+ HNSCC is considered a more immunogenic malignancy due to its viral origin and expression of distinct viral antigens, thus providing an enhanced antigenic repertoire to be recognized by the immune system.

HPV+ HNSCC substantially differs from HPV-negative HNSCC in terms of its subanatomical location, with the vast majority of HPV+ HNSCC tumors occurring in the oropharynx [10,12]. In contrast, HPV-negative HNSCC tumors are mostly located outside the oropharynx and commonly affect the tongue or oral cavity. It is important to highlight that the oropharyngeal areas affected by HPV+ HNSCC such as the tonsils and base of tongue are lymphoid-rich tissues. As a corollary, general comparisons between HNSCC tumors based on HPV status thus not only evaluate the impact of HPV but also the nature of the affected tissue (lymphoid vs. non-lymphoid), unless HPV+ HNSCC tumors are carefully matched with rare HPV-negative tonsilar or base of tongue cancers.

**Figure 1:**
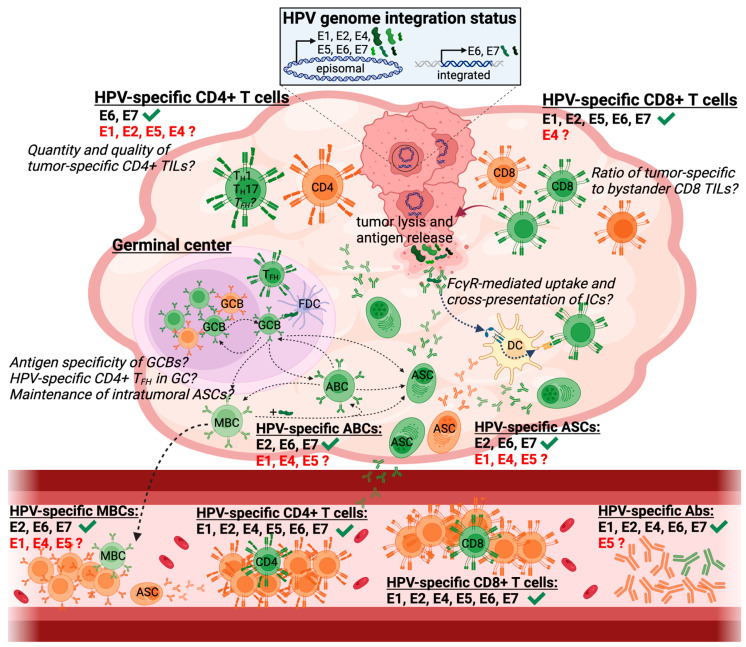
The HPV-specific immune landscape in the tumor and peripheral blood of patients with HPV+ HNSCC. Schematic highlighting HPV-specific immune responses as well as HPV genome integration status and its impact on HPV protein expression. The tumor microenvironment contains germinal center B cells (GCBs), memory B cells (MBCs), activated B cells (ABCs), antibody (Ab)-secreting cells (ASCs), follicular dendritic cells (FDCs), dendritic cells (DCs), and CD8+ T cells (CD8), as well as several CD4+ T helper (T_H_) subsets. Tumor/HPV-specific immune cells are depicted in green and non-tumor-specific bystander cells in orange. Outstanding questions are highlighted in italics. Dashed lines indicate potential interactions/differentiation trajectories. Antigen specificities that have not been experimentally validated in the respective compartment and immune cell subset are highlighted in red. Created with BioRender.com.

Mounting evidence demonstrates that, in HPV-associated cancers such as cervical cancer and HNSCC, the HPV genome can exist in distinct forms: episomal, integrated, or a mixture of both. In most cervical cancer cases, the HPV genome is stably integrated into the host genome [71]. Notably, the linearization of the HPV genome as a result of the integration event most frequently occurs in the coding region for the early gene E2, which is a crucial transcription factor and responsible for tightly regulating the expression of the viral oncogenes E6 and E7 during the viral life cycle [72]. The absence of functional E2 protein, due to HPV genome integration, thus prevents the transcriptional repression of E6 and E7 expression, resulting in excessive cell proliferation and cell transformation. While the integration of the HPV genome in the E2 coding region and concomitant abrogation of functional E2 protein is considered a key event in HPV-mediated oncogenesis, this is not a universal requirement as E2-mediated regulation of E6 and E7 expression can also be impacted by other mechanisms such as methylation of the E2 binding sites in the upstream regulatory region of E6 and E7 [73]. Notably, primary samples of cervical cancer can also contain a mix of integrated and episomal HPV genomes, with episomal forms being lost upon cell culturing [71,74]. However, whereas in more than 80% of cervical cancers the HPV genome is integrated into the host genome [75], the vast majority of HPV+ HNSCC contains episomal HPV genomes, either in a pure episomal form or a mixture of integrated and episomal genomes [16]. This major but not well understood difference between cervical cancer and HNSCC likely has important implications regarding the immunogenicity of these two HPV-driven malignancies. An emerging body of evidence demonstrates that several, if not all, early genes (E1, E2, E4, E5, E6, and E7) are expressed when the HPV genome is episomally maintained [16,76,77]. The expression of additional viral antigens, besides the classical oncoproteins E6 and E7, thus likely endows these cancers with a significantly larger antigenic repertoire (Figure 1), especially when considering that E1 and E2 surpass the relatively small oncoproteins E6 and E7 two- to six-fold in terms of size and thus antigenic information. Hence, the immune response to HPV+ HNSCC containing episomal HPV genomes is expected to be of greater breadth compared to cervical cancers that mostly harbor integrated HPV genomes.

## 3. HPV-Specific Immune Responses in HPV+ HNSCC

The viral origin of HPV+ HNSCC and hence the continuous presence of foreign antigens of defined nature renders HPV+ HNSCC an exquisite malignancy to probe bona fide tumor-specific immune responses in humans. The presence of defined virus-derived tumor antigens further facilitates the development of cost-effective, off-the-shelf therapeutic vaccines for this disease, which is in stark contrast to other non-viral malignancies that require personalized and laborious targeting of private neo-antigens. Importantly, to date, the vast majority of studies analyzing HPV/tumor-specific immune responses in patients with HPV+ HNSCC have focused entirely on peripheral blood. This somewhat paradoxical focus on tumor-specific immune responses in circulation for a solid malignancy such as HPV+ HNSCC is likely due the ease of accessing peripheral blood and independence from a high-volume surgical center required to obtain sufficient fresh tumor tissues for immunological analyses. Here, we will review the immunological studies performed so far and highlight the few studies assessing HPV-specific immune responses in the tumor microenvironment (TME).

### 3.1. HPV-Specific Immune Responses in the Peripheral Blood

#### 3.1.1. HPV-Specific T Cell Responses

The analysis of HPV-specific immune responses in the peripheral blood of patients with HPV+ HNSCC has so far mostly focused on either the enumeration and characterization of HPV-specific T cell responses or HPV-specific antibodies. Studies assessing HPV-specific T cell responses have repeatedly shown that T cell responses against various HPV E proteins are present in most patients with HPV+ HNSCC (Figure 1) [78,79,80,81,82,83]. However, in general, these responses are undetectable directly ex vivo and require extensive in vitro expansion with HPV peptides for at least 1–2 weeks prior to being detectable by standard approaches such as ELISpot, intracellular cytokine staining, or pMHC-I/II tetramer staining [78,79,80,81,82,83]. Notably, CD4+ and CD8+ T cell responses directed against all HPV E proteins (E1, E2, E4, E5, E6, and E7) can be detected in most patients [81]. However, to date, most studies have exclusively focused their analyses on T cell responses against the oncoproteins E6/7 and neglected to assess responses against other HPV E proteins [79,80,82,83]. The exclusive focus of immunological studies on the oncoproteins E6/7 was most likely driven by the now outdated assumption that complete HPV genome integration, akin to cervical cancer, occurs in most HPV+ HNSCC.

While in vitro expansion of antigen-specific T cells with peptides represents a powerful approach to detecting antigen-specific T cell responses, it also has several limitations. First, it is unclear whether the expanded antigen reactivities obtained after prolonged in vitro stimulation with peptide pools accurately reflect their in vivo distribution, as different antigen specificities can exhibit striking differences in their capacity to expand in vitro [84]. Second, while in vitro expansion procedures allow for the greatest sensitivity for detecting antigen-specific T cells, significant phenotypic changes occur during prolonged in vitro stimulation [84], precluding firm conclusions about the in vivo phenotype of these cells. Notably, the fact that circulating HPV-specific T cells expand upon in vitro peptide stimulation, however, suggests that all or at least a fraction of these cells possess proliferative capacity. Thus, while data derived from in vitro expanded T cells can provide clues regarding the fine specificity and proliferative potential of circulating HPV-specific T cells in a given patient, they do not provide any insights into the true in vivo frequency and phenotype of HPV-specific T cells in circulation and, even more important, into the antigen specificity and phenotype of HPV-specific T cells in the TME.

#### 3.1.2. HPV-Specific Humoral Responses

HPV-specific antibodies can be detected in the plasma of most patients with HPV+ HNSCC but are rarely present in individuals without HPV+ HNSCC, including patients with HPV-negative HNSCC (Figure 1) [11,49,85,86,87,88,89,90]. These HPV-specific antibodies target most HPV E proteins, with E6 and E7 being the most widely studied reactivities, akin to the “historic” focus on HPV-specific T cell responses against these antigens.

The presence of HPV E-specific antibodies has been suggested as a sensitive and specific biomarker for early disease detection. Notably, antibodies against HPV E6 are detectable in the blood of patients with HPV+ HNSCC long before clinical diagnosis, with seroconversion being observed on average about 11.5 years, in some patients up to 30 years, prior to diagnosis [49,50]. Based on these data, a recent study estimated the absolute risk of HPV+ HNSCC in HPV E6 seropositive individuals and found the 10-year risk for seropositive males to be between 17% and 27%, with an estimated 30-year risk approaching almost 50% for seropositive males [91]. Efforts are currently ongoing to harness a general, prospective, population-based cohort study, the Hamburg City Health Study, with an overall enrollment goal of 45,000 middle-aged participants (45–74 years of age), to further evaluate HPV-specific antibodies for early disease detection. Notably, an interim analysis of about 4500 serum samples identified several individuals seropositive for HPV E6 and at least one additional HPV E antigen. Three out of nine seropositive individuals who consequently underwent regular head and neck follow-up examinations were diagnosed with HPV+ HNSCC within three to four years of their initial blood draw, further supporting the diagnostic value of HPV serology for early disease detection [92].

In contrast to the accumulating data supporting the use of HPV serology for early diagnosis of HPV+ HNSCC, the predictive value of HPV-specific antibodies for survival and recurrence is still controversial [85,86,93,94,95]. While one study found an association between higher pretreatment E6 antibody titers and increased risk of recurrence [94], two studies reported opposing findings, with E6 seropositivity being associated with improved progression-free survival and reduced risk of locoregional recurrence [85,86]. Furthermore, two studies found no association between pretreatment E6 antibody titers and risk of recurrence [93,95]. However, one of these studies reported significantly higher E6 and E7 antibody titers in recurrent patients during the follow-up period [93], suggesting the continuous maintenance of active B cell responses due to antigen persistence. The reasons for these contradictory findings are likely to be manifold. All studies analyzed rather small cohorts of patients (n ≤ 115), which combined with the low recurrence rate of HPV+ HNSCC resulted in almost all studies in a very low number of recurrent cases. Notably, the study by Spector et al. [93] contains, to date, the largest number of recurrent cases (n = 22) and found no association between pretreatment antibody titers and risk of recurrence. The cohorts of the above-mentioned studies also varied substantially in terms of treatment modalities. One study focused exclusively on chemoradiation [93], whereas the other studies included patients receiving a wide range of treatment modalities. The studies can further be divided based on their approach of detecting HPV-specific antibodies, with two studies employing a pan-immunoglobulin (Ig) approach (IgA/G/M) [85,95] and three studies focusing on IgG antibodies [86,93,94]. Notably, we have recently shown that HPV-specific plasma antibodies in HPV+ HNSCC patients mostly consist of the IgG isotype, with negligible IgM contribution [11]. However, a subset of patients exhibits sizeable IgA responses, prompting further investigations into the predictive value of distinct Ig isotypes. Overall, additional large-scale studies are required to ultimately answer the question whether HPV-specific antibodies can be employed as prognostic biomarkers for HPV+ HNSCC.

Although HPV-specific antibodies have been extensively studied for their value as early diagnostics and their predictive value for survival and recurrence, it is currently not known whether HPV-specific antibodies play an active role in anti-tumor immunity or rather just represent a surrogate for cellular cytotoxicity and associated tumor antigen release. While it is unlikely, due to the intracellular localization of HPV E proteins, that HPV-specific IgG antibodies exhibit direct anti-tumor effects through antibody-dependent cellular cytotoxicity (ADCC) or phagocytosis (ADCP), these antibodies could potentially contribute to the maintenance of cytotoxic CD8+ T cell responses. Upon tumor cell lysis and antigen release, HPV-specific antibodies could bind to their respective target antigens, form immune complexes, mediate FcγR-dependent antigen uptake, and thus enhance cross-presentation of HPV antigens on professional antigen-presenting cells [96]. Importantly, previous studies evaluating HPV-specific IgG antibodies did not assess the IgG subclass composition, which allows for the diversification of IgG effector functions. In humans, four IgG subclasses (IgG1–4) exist, which differ dramatically in their affinity for distinct FcγRs and thus their ability to engage various effector cells for downstream IgG effector functions [97,98]. Notably, we recently showed that the vast majority of HPV-specific plasma antibodies are IgG1 [11], a highly active IgG subclass that can efficiently trigger activating FcγRs and IgG effector functions such as ADCC or ADCP. However, a subset of patients also exhibited substantial IgG2 and IgG4 responses, which are commonly associated with low or absent IgG effector functions. Our findings might thus at least partially explain the discordant findings in the predictive value of HPV-specific antibodies [85,86,93,94,95,99], as none of previous studies assessed IgG subclasses to account for their divergent IgG effector functions [85,86,93,94,95].

In addition to the presence of HPV-specific antibodies in circulation, we recently demonstrated the presence of HPV-specific memory B cells (MBCs) in the peripheral blood of patients with HPV+ HNSCC [11]. MBCs are an important component of the humoral immune system and together with antibody-secreting, long-lived plasma cells provide two complementary B cell memory “walls” to mediate protection from pathogens [100]. In contrast to long-lived plasma cells that constantly secrete large amounts of antibodies, mostly reside in the bone marrow, are quiescent, and represent a terminally differentiated cell type, MBCs do not produce antibodies, are found in circulation, undergo slow homeostatic proliferation to maintain their numbers, and can quickly differentiate into antibody-secreting cells as well as reenter new germinal center reactions upon reencounter of their cognate antigen [101]. On average, HPV E2-, E6-, and E7-specific MBCs each accounted for about 0.2% of circulating IgG+ MBCs in patients with HPV+ HNSCC [11]. In comparison, influenza-specific IgG+ MBCs accounted for roughly 1% of the total IgG+ MBC pool, consistent with previous reports in healthy individuals [102]. These data demonstrate that HPV-specific MBCs, while representing not the most dominant antigen reactivity in the MBC pool, are readily detectable and account for a sizeable fraction of the IgG+ MBC pool in patients with HPV+ HNSCC.

Chronic inflammation and antigenic stimulation have been shown to contribute to the development of atypical MBCs, a distinct subset of MBCs that is characterized by impaired functionality and recall potential [103]. Notably, HPV-specific IgG+ MBCs detected in patients with HPV+ HNSCC were functional [11], as the experimental approach for their enumeration relied on their reactivation and differentiation into antibody-secreting cells prior to detection [104,105,106]. However, whether the HPV-specific IgG+ MBCs we detected only represent a functional subset of the total HPV-specific IgG+ MBCs pool that might also contain atypical, dysfunctional MBCs requires further investigation.

Overall, these studies demonstrate that HPV-specific immune responses in the peripheral blood of patients with HPV+ HNSCC differ quite dramatically between the humoral and the cellular arms of the adaptive immune system. Thus, while HPV-specific B cell responses in the form of antibodies and MBCs are readily detectable in circulation, HPV-specific T cell responses are, although present, quite rare and require in vitro expansion prior to analysis, thus hampering their analysis.

### 3.2. Intratumoral Immune Responses in HPV+ HNSCC

While HPV-specific immune responses in the circulation of patients with HPV+ HNSCC have been relatively well studied, little work has been performed regarding the magnitude, breadth, and differentiation state of HPV-specific tumor-infiltrating lymphocytes (TILs). Notably, several studies assessed immune infiltrates in HPV+ HNSCC tumors and contrasted them with HPV-negative HNSCC, demonstrating important key differences in the composition of the TME between these two distinct diseases [107,108,109,110,111,112,113,114]. Overall, HPV+ HNSCC exhibits several features associated with a more inflamed or “hotter” immune microenvironment such as increased B cell infiltrates and higher frequencies of PD-1+ CD8+ TILs, T helper type 1 (T_H_1) CD4+ T cells, T_H_17 CD4+ T cells, and T follicular helper (T_FH_) CD4+ T cells (Figure 1). Furthermore, the gene expression profiles of tumor-infiltrating B cells, CD4+ T cells, and myeloid cells demonstrate marked differences between HPV+ and HPV-negative tumors [107], further underlining that these two malignancies are immunologically highly distinct.

#### 3.2.1. Intratumoral Lymphoid Structures

The increased presence of intratumoral B cells is one of the hallmarks distinguishing HPV+ from HPV-negative HNSCC [107,108,110,114]. Importantly, these two distinct malignancies also differ substantially in the composition of intratumoral B cells, with HPV+ tumors containing more germinal center (GC) B cells [107,110]. These intratumoral GC B cells expressed canonical GC B cell genes such as *BCL6*, *AICDA*, and *TCL1A*, and were located in bona fide GCs or structures resembling GCs [11,110]. Notably, consistent with their role in maintaining GC reactions [115], T_FH_ CD4+ T cells were also found to be more prevalent in HPV+ HNSCC [107,110], suggesting ongoing GC reactions in the TME (Figure 1). Overall, B cells in HPV+ HNSCC tumors were preferentially localized in distinct lymphoid structures ranging from unstructured aggregates within the tumor stroma to fully developed GCs [11,110].

Tertiary lymphoid structures (TLSs), which are defined as de-novo generated lymphoid structures within non-lymphoid tissues and consist to a substantial degree of B cells, have been associated with improved overall survival as well as clinical responses to immunotherapy in many cancer types [110,116,117,118]. Importantly, TLSs can exist along a wide spectrum of maturation states ranging from lymphoid aggregates to fully defined secondary follicles with GCs, with the most mature TLS form exhibiting the greatest association with improved outcomes. Due to this association, intensive efforts are currently ongoing to elucidate the exact steps leading to TLS formation, with the ultimate goal of harnessing TLSs for improved responses to immunotherapy [119]. Notably, the presence of intratumoral GC B cells and structures resembling TLSs has also been associated with improved survival in both HPV+ and HPV-negative HNSCC [110]. However, it is important to note that, due to preferential occurrence of HPV+ HNSCC on the tonsils or base of tongue, both of which are lymphoid-rich tissues, the lymphoid structures observed in HPV+ HNSCC tumors cannot be considered “classic” TLSs (i.e., de novo generated lymphoid structures in non-lymphoid tissues). While it is plausible that some of the observed structures in HPV+ HNSCC tumors are de novo generated, markers faithfully distinguishing these potentially de novo generated structures from remnant lymphoid structures that have been engulfed by the tumor are lacking. Thus, although it might be a semantic argument, it is important to consider the lymphoid nature of the tissues affected by HPV+ HNSCC when analyzing TLS-resembling lymphoid structures in this malignancy and attempting to translate findings to other malignancies occurring in non-lymphoid tissues.

#### 3.2.2. HPV-Specific TIL Responses

The analyses of immune infiltrates yielded important insights into the TME of HPV+ HNSCC, demonstrating an overall more immunologically active environment compared to HPV-negative HNSCC as highlighted by an increased presence of CD8+ T cells [108,120,121]. However, the antigen specificity of TILs in HPV+ HNSCC was not assessed in most studies. Notably, in various malignancies a sizeable fraction, if not the majority, of intratumoral CD8+ T cells are bystander cells that are not tumor-specific but directed against common human pathogens such as herpesviruses and influenza viruses [122,123,124,125,126,127]. Bystander CD8+ T cells often exhibit phenotypic traits commonly associated with T cell exhaustion such as expression of the inhibitory receptor PD-1, thus “disguising” themselves as exhausted T cells at first glimpse despite being highly functional memory T cells [99,128]. Hence, the presence of T cells in the TME should not be confused or equated with their tumor specificity. Importantly, while bystander infiltration has been assessed in various malignancies such as melanoma, lung, and colorectal cancer, no dedicated studies have been performed in HPV+ HNSCC. However, such studies would provide important insights into intratumoral bystander recruitment by contrasting previous findings with a primary tumor that predominantly occurs in lymphoid tissues and is of viral origin. While the extent of bystander recruitment in HPV+ HNSCC is unknown and needs to be experimentally assessed, we would anticipate, due to the lymphoid character of the affected tissue, a considerable proportion of bystander T cells to be present. Overall, a better understanding of the bystander populations and their antigen specificities might further open additional therapeutic venues by harnessing their functional potential for improved tumor control [99,124,129,130].

As mentioned above, relatively little work has been performed regarding HPV-specific TILs in HPV+ HNSCC. Prior works showed the presence of intratumoral HPV E6- and E7-specific CD4+ and CD8+ T cells, which predominantly secreted IFN-γ but also a substantial amount of IL-17, suggesting the intratumoral presence of HPV-specific T_H_1 and T_H_17 responses in HPV+ HNSCC (Figure 1) [83,131,132]. However, additional information about the phenotypic and transcriptional landscape of HPV-specific TILs required to assess the differentiation state of these cells and their potential responsiveness to various treatment modalities has been lacking. The analysis of HPV-specific TIL responses has been mostly restricted to the viral oncoproteins E6 and E7, as other viral antigens were thought not to be expressed due to complete HPV genome integration, which, however, only occurs in a minority of HPV+ HNSCC tumors as highlighted by recent studies [16,76,77]. We recently reported the presence of HPV-specific CD8+ T cells in the TME of patients with HPV+ HNSCC, demonstrating that a substantial proportion of CD8+ TILs are directed against HPV antigens other than the oncoproteins E6 and E7. Using peptide-MHC-I tetramers that allow for the physical detection of antigen-specific CD8+ T cells, we showed that in some HPV+ HNSCC tumors up to 10% of CD8+ TILs recognized a given epitope [78]. Surprisingly, most CD8+ T cell epitopes discovered in our study were derived from HPV E2 and E5, which also encompassed the most immunodominant responses, with little to no reactivity detected against HPV E6 and E7. Notably, a recent study identified CD8+ TIL responses against HPV E1, E2, and E6 [133], further supporting the notion that “non-classical” HPV antigens such as E1, E2, E4, and E5 are targeted by a substantial portion of HPV-specific CD8+ TILs and should thus not be neglected in the development of novel therapeutic interventions for HPV+ HNSCC. Importantly, our findings also confirmed previous studies demonstrating that HPV-specific CD8+ T cells are virtually undetectable in the peripheral blood of patients with HPV+ HNSCC [79,80,81,82,83], despite individual epitope-specific responses accounting for up to 10% of CD8+ T cells in the TME [78]. Overall, these data highlight that HPV-specific T cell responses target several HPV antigens and are highly localized to the TME.

HPV-specific CD8+ T cell responses in HPV+ HNSCC tumors were not only characterized by a high degree of tissue retention but also exhibited a striking degree of oligoclonality, with the four most prevalent clonotypes accounting for more than 50% of the cells responding to a given epitope in most patients [78]. In some patients with particularly immunodominant HPV-specific CD8+ TIL responses, individual clonotypes made up for more than 5% of all CD8+ TILs, further highlighting the high degree of oligoclonality, which is likely driven by extensive and prolonged antigen exposure. Importantly, HPV-specific CD8+ T cell responses and their clonotypic composition were comparable in patient-matched primary tumor and metastatic lymph node samples, suggesting that different HPV+ HNSCC tumor sites exhibit similar immune environments and immune pressure [78].

**Figure 2:**
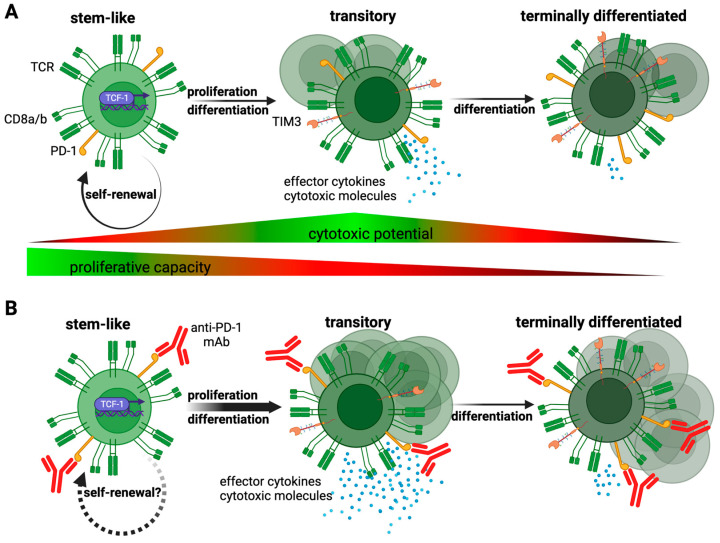
Differentiation states of HPV-specific PD-1+ CD8+ T cells in HPV+ HNSCC tumors. (**A**) Lineage relationship of HPV-specific CD8+ T cell subsets found in primary tumors and metastatic lymph nodes of patients with HPV+ HNSCC. Cytotoxic potential and proliferative capacity are indicated. (**B**) PD-1 pathway blockade increases proliferation and differentiation of stem-like CD8+ T cells into transitory cells and abrogates PD-1-mediated inhibition of transitory and terminally differentiated cells, resulting in increased secretion of effector cytokines and cytotoxic molecules. Based on data from preclinical mouse models. Dashed arrow indicates the unclear role of PD-1 pathway blockade onto self-renewal rate of stem-like cells. Created with BioRender.com.

HPV-specific CD8+ TILs in primary tumors and metastatic lymph nodes of patients with HPV+ HNSCC uniformly expressed high levels of the inhibitory receptor PD-1 and the transcription factor TOX [78], which are both commonly associated with T cell exhaustion. A detailed analysis of HPV-specific CD8+ TILs from both tumor sites revealed a marked heterogeneity of these exhausted CD8+ T cells, with three distinct clusters being present in all examined samples: stem-like, transitory, and terminally differentiated cells (Figure 2A). Stem-like CD8+ T cells, often also referred to as progenitor exhausted, are characterized by co-expression of PD-1, TOX, and TCF-1, an important transcription factor encoded by *TCF7* and crucial for imparting the stem-like properties onto this subset [134,135,136,137,138,139]. Notably, stem-like CD8+ T cells possess the ability to self-renew, exhibit substantial proliferative potential, lack cytotoxic capacity but can give rise to a more differentiated, cytotoxic progeny, and are essential for maintaining CD8+ T cell responses during conditions of antigen persistence such as chronic viral infections and cancer. Importantly, this subset has been shown to be responsible for the proliferative burst of CD8+ T cells upon PD-1 pathway blockade in several preclinical mouse models (Figure 2B) [134,135,136,137,138,139], and its intratumoral presence has been associated with improved outcomes and responsiveness to immune checkpoint blockade (ICB) in several malignancies [138,140,141]. Transitory cells are recently differentiated cells and likely exhibit the highest cytotoxic capacity among exhausted CD8+ T cells [142,143]. They express high levels of effector molecules such as granzymes and perforin, and relatively low levels of additional co-inhibitory receptors such as CD39 when compared to terminally differentiated CD8+ T cells, which represent the most dysfunctional cells [142,143]. Notably, the three clusters of HPV-specific CD8+ TILs exhibited a substantial degree of clonal overlap, with individual clonotypes being present in all three differentiation states [78]. These data thus strongly support a distinct lineage relationship model in which HPV-specific stem-like CD8+ T cells give rise to the more differentiated subsets (Figure 2A), akin to the experimentally validated lineage relationship of exhausted T cells in preclinical mouse models [134,136]. It is important to highlight that HPV-specific PD-1+ TCF-1+ CD8+ T cells in patients with HPV+ HNSCC not only phenotypically resembled stem-like CD8+ T cells but indeed possessed stem-like capabilities [78]. HPV-specific stem-like CD8+ T cells proliferated and differentiated into a more effector-like state, characterized by upregulation of granzyme B and TIM3, when stimulated in vitro with their cognate antigen, highlighting the therapeutic potential of targeting this unique CD8+ T cell subset through ICB and/or therapeutic vaccination. Overall, these data demonstrate that HPV-specific CD8+ T cells responsive to ICB are abundantly present in the TME of HPV+ HNSCC, suggesting that this malignancy contains the cellular machinery necessary for response to ICB in situ.

#### 3.2.3. HPV-Specific Intratumoral B Cell Responses

Increased frequencies of B cells as well as the presence of GC B cells and physical GCs are characteristics of HPV+ HNSCC tumors [107,108,110,114]. However, little is known about the antigen specificity of intratumoral B cells in HPV+ HNSCC. We recently demonstrated the intratumoral presence of HPV-specific B cells in primary tumors and metastatic lymph nodes [11]. Antibody-secreting cells (ASCs) producing IgG antibodies specific for HPV E2, E6, and E7 were readily detectable in the vast majority of HPV+ HNSCC tumors but absent in HPV-negative tumors. HPV-specific IgG+ ASCs accounted for 0.1–20% of all intratumoral IgG+ ASCs, with E2 being the most dominant target, similar to our findings regarding the intratumoral CD8+ T cell response [78]. Notably, HPV-specific IgG+ ASC responses in primary tumors correlated with responses observed in patient-matched metastatic lymph nodes, suggesting that ongoing anti-tumor responses are mirrored between these two tumor sites. Furthermore, we observed a positive correlation between intratumoral HPV-specific IgG+ ASCs and HPV-specific plasma IgG titers, suggesting that HPV-specific plasma IgG antibodies can provide insights into the intratumoral HPV-specific ASC response in the absence of available tumor tissue [11]. In contrast to the abundance of HPV-specific IgG+ ASCs in the TME, no HPV-specific ASCs were detected in the peripheral blood, indicating a highly localized ASC response akin to the HPV-specific T cell response in HPV+ HNSCC.

Notably, the presence of HPV-specific ASCs in the TME was not driven by indiscriminate, inflammation-associated recruitment and differentiation of circulating MBCs. Influenza-specific IgG+ ASCs were rarely detectable intratumorally, while influenza-specific IgG+ MBCs vastly outnumbered HPV-specific IgG+ MBCs in the circulation of patients with HPV+ HNSCC [11]. These findings suggest that, among intratumoral IgG+ ASCs in HPV+ HNSCC, bystander responses directed against common human pathogens such as influenza are relatively rare and that most IgG+ ASCs might be directed against tumor antigens, which is in stark contrast to the ample presence of bystander CD8+ TILs in most malignancies [122,123,124,125,126,127]. However, additional studies are required to support this notion, as even in patients exhibiting the highest intratumoral HPV E2-, E6-, and E7-specific ASC responses, the assessed HPV specificities accounted for less than 30% of IgG+ ASCs, raising important questions about the specificity of the vast majority of intratumoral ASCs.

Besides ASCs, the TME of HPV+ HNSCC also contains additional active B cell subsets that are identified by elevated expression of the transferrin receptor CD71: activated B cells (ABCs), GC B cells, and a small cluster of transitory cells sharing transcriptional similarities with both GC B cells and ASCs [11]. The antigen specificity of ABCs was assessed by flow cytometric staining with a fluorescently labeled HPV E2 antigen probe and subsequently confirmed by generation of human monoclonal antibodies (hmAbs). The generation of hmAbs not only yielded useful reagents to assess the role of HPV proteins in the viral life cycle [144] but also revealed a striking degree of somatic hypermutation (SHM) in the antibody-encoding genes of intratumoral HPV-specific ABCs. Notably, the degree of SHM in HPV-specific ABCs was substantially greater than in human B cells elicited by various vaccinations or acute viral infections [145,146,147,148,149,150], suggesting that the observed intratumoral HPV-specific B cell responses represent actively ongoing responses driven by continuous antigen exposure. ABCs are B cells that recently encountered their cognate antigen and do not secrete antibodies [145]. Furthermore, ABCs are distinct from MBCs by elevated expression of several genes related to antigen presentation such as MHC-II, suggesting that they might actively present antigens to intratumoral CD4+ T cells. While direct interactions between HPV-specific ABCs and CD4+ TILs, which have been scarcely studied regarding their antigen specificity in HPV+ HNSCC [131,133], are likely to occur in the TME, direct interactions between HPV-specific B cells and CD8+ T cells are, despite similar antigen reactivity patterns [11,78], unlikely as dendritic cells (DCs) and not B cells are in general considered the major cell type capable of antigen cross-presentation [151]. Although B cells might not directly present antigens to CD8+ T cells, the production of HPV-specific antibodies might still indirectly contribute to the cross-presentation of HPV antigens by enhancing uptake and processing of HPV antigens in the form of immune complexes and subsequent cross-presentation by DCs [96], providing a potential mechanistic link explaining the comparable antigen reactivity patterns between HPV-specific CD8+ T cells and B cells in the TME (Figure 1).

## 4. Implications for Immunotherapy

The overall “hot” immune microenvironment in HPV+ HNSCC tumors combined with the presence of conserved, virus-derived antigens that are targeted by both intratumoral T and B cells renders this disease a prime candidate for immunotherapeutic interventions. Notably, current de-escalation strategies attempt to incorporate immunotherapeutic treatment modalities with the overall goal of reducing morbidities associated with traditional treatments such as chemotherapy and radiation, while maintaining high cure rates [59,60].

### 4.1. Immune Checkpoint Blockade

ICB has transformed the clinical management of several malignancies, and several inhibitors of the PD-1 signaling pathway have obtained FDA approval over the past decade, including for the treatment of HNSCC [59,152]. However, despite the ample presence of HPV-specific B and T cells in the TME and expression of viral antigens by the tumor, response rates to ICB have been rather disappointing and did not exceed 30% in patients with HPV+ HNSCC [59,153]. This is especially surprising as HPV+ HNSCC tumors not only contain HPV-specific CD8+ T cells but also a substantial number of stem-like CD8+ T cells [78], which have been linked to responsiveness to ICB in preclinical mouse models as well as several clinical studies [135,136,137,138,139,140,141]. Notably, the substantial infiltrations of HPV-specific CD8+ T cells, including the stem-like subset, were observed in treatment-naïve patients undergoing surgical resection as first-line treatment [78]. In contrast, ICB has so far only been assessed in the recurrent setting, in which patients with HPV+ HNSCC had previously received several rounds of conventional treatments such as chemotherapy and radiation. These treatment modalities might have substantially reduced or potentially even completely abrogated intratumoral HPV-specific CD8+ T cell responses, thus hampering subsequent responsiveness to ICB. An alternative explanation for the limited ICB responses in patients with HPV+ HNSCC might be the presence of additional, unknown immunoregulatory mechanisms preventing the proliferation and differentiation of HPV-specific stem-like CD8+ T cells or the execution of sufficient effector functions of their cytotoxic progeny upon ICB. Further studies are required to assess how the TME and especially intratumoral T cell responses of patients with HPV+ HNSCC evolve in response to conventional treatment modalities in terms of antigen specificity and differentiation state.

### 4.2. Therapeutic Vaccines

The presence of a defined set of conserved antigens of foreign/viral nature in HPV+ HNSCC renders this malignancy an excellent candidate for therapeutic vaccination approaches. Notably, multiple therapeutic vaccine platforms including peptide-, nucleic-acid-, and vector-based vaccines are currently being investigated for the treatment of several HPV-associated malignancies including HPV+ HNSCC [154]. While the employed vaccine platforms vary widely, they are all exclusively focused on eliciting immune responses against the viral oncoproteins E6 and E7, thus not taking advantage of the full antigenic breadth of most HPV+ HNSCC tumors, which express virtually all HPV E proteins due to episomal maintenance of the HPV genome [16,76,77].

Therapeutic vaccines can improve anti-tumor CD8+ T cell responses, in general, through two distinct mechanisms: (i) either by de novo priming CD8+ T cells that were not recruited into the initial response due to inefficient or absent presentation of their respective target peptides on professional antigen-presenting cells or (ii) by re-priming/stimulation of antigen-experienced CD8+ T cells to undergo further proliferation. Although definitive experimental data in humans are lacking, data from preclinical mouse models demonstrate that therapeutic vaccines can exert detectable therapeutic effects through the stimulation of pre-existing, exhausted CD8+ T cells [155]. These responses are most likely mediated by re-priming of stem-like CD8+ T cells, the only exhausted T cell subset with proliferative potential, through the simultaneous provision of cognate antigen and co-stimulatory signals such as CD28, which has been shown to be crucial for the proliferative burst of exhausted CD8+ T cells in response to ICB [156].

The limited clinical efficacy of therapeutic vaccines for HPV+ HNSCC, especially in the monotherapy setting, can also be explained by the fact that, even if anti-tumor responses are elicited, the primed or re-primed CD8+ T cells will eventually encounter negative immunoregulatory signals in the TME such as PD-L1 and other immunoregulatory cell subsets such as regulatory T cells and myeloid suppressor cells, thus blunting their activity [157]. Notably, while therapeutic vaccines employing synthetic long peptides (SLPs) of HPV E6 and E7 elicit robust HPV-specific T cell responses in patients with premalignant lesions and cervical cancer, their clinical efficacy as monotherapy is limited to the treatment of premalignant anogenital lesions, with clinical responses in about 50% of individuals [158,159,160]. Preclinical data demonstrate that, while therapeutic vaccines as monovalent therapies can have modest impacts on antigenic burden, concomitant ICB exerts synergistic effects and results in efficient rejuvenation of exhausted CD8+ T cell responses and striking therapeutic effects [155]. In line with this, a recent trial assessing the combination of HPV SLPs and PD-1 pathway blockade showed promising results in patients with HPV+ HNSCC, with overall response rates of around 33% [161]. Furthermore, the combination of HPV SLPs and chemotherapy to ablate immunosuppressive myeloid cells induced robust T cell responses, which were associated with prolonged survival of patients with cervical cancer [162,163].

Overall, these data suggest that inclusion of additional HPV antigens, besides the classical HPV oncoproteins E6 and E7, into therapeutic HPV vaccines and their combination with ICB or other immunomodulatory interventions will unleash anti-tumor immune responses of maximal breadth, magnitude, and functionality in HPV+ HNSCC.

### 4.3. Adoptive Cell Therapies

Adoptive cell therapies (ACTs) are another promising immunotherapeutic treatment modality for HPV+ HNSCC. Overall, two distinct adoptive cell therapy approaches have been evaluated for their efficacy against HPV-associated cancers in phase I/II studies: infusion of TIL products that were enriched for reactivity against HPV E6 and E7 [164,165,166], or infusion of T cells genetically engineered to express a T cell receptor (TCR) directed against either an HPV E6- or E7-derived peptide presented by HLA-A*02:01 [167,168,169]. Both ACT approaches showed promising results with objective responses in a substantial fraction of patients, with some patients even exhibiting complete regressions. A major limitation of currently employed TCR-modified ACTs, however, is that they so far exclusively target HPV E antigens restricted by HLA-A*02:01. While HLA-A*02:01 is by far the most prevalent HLA allele in Caucasians, the currently available TCR products do not benefit the vast majority of the population. Notably, we recently identified several TCRs recognizing two immunodominant HPV E2 epitopes presented by HLA-A*01:01 [78], thus expanding the repertoire of potential TCRs for ACTs not only in terms of antigenic breadth but also HLA restriction. Overall, TCR-engineered ACTs are a promising treatment modality, and their further development and broader applicability will benefit from future studies on HPV-specific intratumoral CD8+ T cells by yielding novel TCRs directed against additional epitopes restricted by a wider range of HLAs.

## 5. Conclusions

Substantial progress has been made over the past few decades in the area of HPV+ HNSCC, resulting in a better understanding of the genetic and immunological landscape of HPV+ HNSCC. However, there are still substantial gaps in our knowledge regarding the immunological landscape of this disease. In particular, the antigen specificity and differentiation states of tumor-specific B and T cells within the TME are not well understood. Some of the outstanding questions are: What are the immunodominant antigens recognized by intratumoral HPV-specific B and T cells? Do the differentiation states of TILs specific for distinct HPV antigens differ, with some reactivities being more prone to terminal exhaustion? Is there an interplay between humoral and cellular HPV-specific TILs? If so, what is the mechanistic basis, and can it be harnessed to develop novel therapeutic approaches and improve treatment outcomes?

A better understanding of the immunological landscape will crucially inform the development of novel therapeutic approaches to increase cure rates while simultaneously reducing treatment-associated morbidities. Importantly, the insights gained from studying anti-tumor responses in HPV+ HNSCC will also substantially improve our general understanding of tumor-specific immune responses in humans as this malignancy, due to the expression of distinct virus-derived tumor antigens, can serve as a unique model enabling the study of bona fide tumor-specific B and T cell responses in humans.
